# Cannabis related side effects in otolaryngology: a scoping review

**DOI:** 10.1186/s40463-021-00538-6

**Published:** 2021-09-27

**Authors:** Jobanjit S. Phulka, Joel W. Howlett, Amanda Hu

**Affiliations:** Division of Otolaryngology – Head and Neck Surgery, Department of Surgery, 4th Floor, Gordon and Leslie Diamond Health Care Center, University of British Columbia, 2775 Laurel Street, Vancouver, BC V5Z1M9 Canada

**Keywords:** Head and neck, Voice, Hearing loss, Sinusitis, Cannabis, Adverse effects, Scoping review

## Abstract

**Background:**

Cannabis has been rapidly legalized in North America; however, limited evidence exists around its side effects. Health Canada defines side effect as a harmful and unintended response to a health product. Given drug safety concerns, this study’s purpose was to review the unintended side effects of cannabis in otolaryngology.

**Methods:**

The Preferred Reporting Items For Systematic Reviews and Meta-analysis extension for Scoping Reviews (PRISMA-ScR) protocol was used to conduct a scoping review of the MEDLINE, EMBASE, CINAHL, and CENTRAL databases. (PROSPERO: CRD42020153022). English studies in adults were included from inception to the end of 2019. In-vitro, animal, and studies with n < 5 were excluded. Primary outcome was defined as unintended side effects (defined as any Otolaryngology symptom or diagnosis) following cannabis use. Oxford Centre for Evidence-Based Medicine: Levels of Evidence and risk of bias using the Risk of Bias in randomized trials (RoB 2) and Risk of Bias in Non-Randomized Studies of Interventions (ROBINS-I) tools were assessed.. Two authors independently reviewed all studies; the senior author settled any discrepancies.

**Results:**

Five hundred and twenty-one studies were screened; 48 studies were analysed. Subspecialties comprised: Head and Neck (32), Otology (8), Rhinology (5), Airway (5), Laryngology (1). Cannabis use was associated with unintended tinnitus, vertigo, hearing loss, infection, malignancy, sinusitis, allergic rhinitis, thyroid dysfunction, and dyspnea. About half (54.1%) of studies showed increased side effects, or no change in symptoms following cannabis use. Oxford Levels of Evidence was 2–4 with substantial heterogeneity. Risk of bias assessment with RoB2 was low to high and ROBINS-1 was moderate to critical.

**Conclusion:**

This was the first comprehensive scoping review of unintended side effects of cannabis in Otolaryngology. The current literature is limited and lacks high-quality research Future randomized studies are needed to focus on therapeutic effects of cannabis in otolaryngology. Substantial work remains to guide clinicians to suggest safe, evidence-based choices for cannabis use.

**Graphic abstract:**

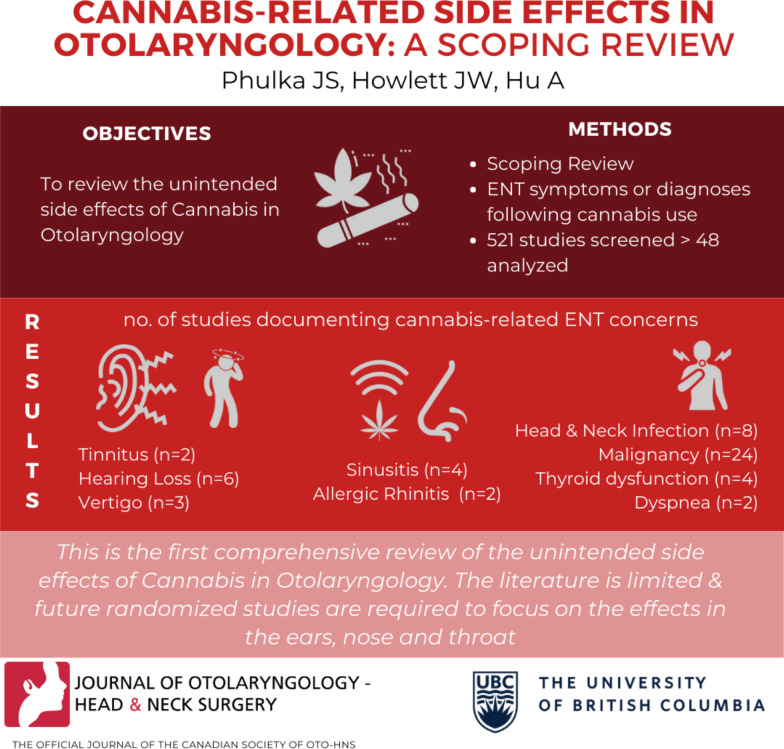

## Introduction

Cannabis is one of the most commonly used drugs in North America. Nearly half of the American population has used cannabis at least once in their lifetime, with approximately 9% being current users [1]. Evolving societal perception has rapidly driven the legalization of cannabis, which has been structured to regulate it’s production and sales, while promoting safe consumption [2]. Legalization has led to a perceived reduction in harm, which has been associated with an increased prevalence of cannabis consumption from 2002 to 2014 [2]. Evidence based research has followed this movement, yet a paucity of data concerning the side effects of this drug remains.

Cannabis is derived from a flowering plant. There are three main forms of cannabis, based on the part of the plant that the drug is produced from: marijuana, hashih, and hash oil [3]. Marijuana is the least potent form and manufactured from the dried flowers and leaves. Hashish is manufactured from the resin or secreted gum of the plant. Hash oil is the most potent and manufactured from the thick oil obtained from hashish [4]. There are three routes of delivery of this drug: oral, dermal, and inhaled. Smoking the drug via the inhaled route is the most popular due to the quick onset of action [4].

Health Canada defines a side effect as “a harmful and unintended response to a health product” [5]. Pharmacists distinguish the term “side effect” from “adverse event” [6]. The latter is an undesired occurrence that results from taking a medication correctly. Side effect occurs when the medication is administered regardless of the dose. Side effect is the more accurate term for this study since the dosage of cannabis was not reliable, especially before its’ legalization. A review from the *New England Journal of Medicine* reported adverse health effects of cannabis to be as high as 50% [7]. This list included: addiction, abnormal brain development, progression to use of other drugs, schizophrenia, depression, anxiety, chronic bronchitis, and lung cancer. Health Canada recognizes the importance of side effects and encourages the reporting of side effects on their website with a special section for cannabis products [8]. The aforementioned list focused on psychiatry and respirology side effects of cannabis. Acute side effects of cannabis in otolaryngology may include type 1 hypersensitivity reactions, cough, rhinosinusitis, laryngopharyngitis, xerostomia, and altered neurotologic function. Chronic usage may result in periodontal disease, voice changes, impaired regulation of cell cycle, apoptosis, and cellular migration, potentially increasing the risk of head and neck malignancy [9]. Prior reviews have investigated cannabis use concerning Otolaryngology, but have been limited to the oncology and laryngology literature [4, 10]. The purpose of this scoping review wass to understand the safety profile of cannabis and how drug use in adults has the potential for unintended side effects related to otolaryngology pathologies. The intention is to be comprehensive throughout all subspecialties of Otolaryngology, and to provide clinicians with knowledge to help patients to make safe, evidence-based choices around the use of cannabis.

## Methods

This scoping review was carried out according to a review protocol that has been published in the Prospective Register of Systematic Reviews (PROSPERO Registration number: CRD42020153022). A systematic review was the initial intention for this study, but the heterogeneity of the literature and lack of high-quality evidence precluded this, and therefore a scoping review was more appropriate. The reporting of this scoping review was conducted in accordance with the Preferred Reporting Items For Systematic Reviews and Meta-analysis extension for Scoping Reviews (PRISMA-ScR) statement [11]. The quality of the literature was assessed with the Oxford Centre for Evidence-Based Medicine: Levels of Evidence [12].

### Data sources and search strategy

A literature search was performed in electronic databases, including MEDLINE, EMBASE, CINAHL, and CENTRAL databases of the Cochrane Library from inception through October 1, 2019. The search strategy was developed with assistance from a medical librarian. Search functions were designed to incorporate two subsections by [AND] Boolean operators. Subsections contained MeSH and field-designated search terms for otolaryngology related diseases and for cannabis. A cannabis search hedge was employed to identify both formal and informal terms for cannabis in the literature [13]. Additionally, reference lists from previously published reviews were screened for articles not identified in the initial search. Detailed search strategies are reported in “Appendix A”.

### Study selection

All articles identified via the literature search were exported to Covidence (Veritas Health Innovation Ltd., Melbourne, Australia), a systematic review management software. Study selection was independently undertaken by two authors (J.P.& J.H.) with discrepancies being resolved by consensus with the senior author (A.H.). Inclusion criteria included: (1) English language study, (2) adult population (≥ 18 years old), (3) sample size ≥ 5, (4) clinical study, (5) study subjects report use or were exposed to cannabis, and (6) report of otolaryngology related side effects (symptom or diagnosis) following cannabis use that were unintended. Of note, no pediatric studies were included in this scoping review. Most legislation for legal cannabis prohibits consumption in the pediatric population. The exclusion criteria included: (1) in-vitro or animal study and (2) inappropriate study or publication type (e.g. systematic review, literature review, or book chapter). Additionally, side effects involving the lower airways were excluded as this is generally considered outside of the scope of an Otolaryngologist, and primarily managed by Pulmonology Medicine. The areas of wound healing, analgesia and pain were excluded as they are non-specific to the head and neck. Lastly, the purpose of this scoping review was not to evaluate the therapeutic indications of cannabis in otolaryngology, but to evaluate the unintended otolaryngologic side effects of consuming cannabis recreationally or medically.

### Main outcome

This study used Health Canada’s definition of side effect [5]. The primary study concept was to examine unintended Otolaryngology related side effects following any level of cannabis exposure. Otolaryngology related side effects were defined as a pathologic diagnosis or symptom commonly treated by an Otolaryngologist.

### Data extraction

Data extraction was conducted by a single author (J.P.) and reviewed by a second author (J.H.). Information extracted from each study included: title, first author, year of publication, study design and objectives, characteristics of study participants, intervention(s) and control(s), tobacco use, cannabis consumption and amount (e.g. joint year), primary study outcomes and main findings including otolaryngology related side effects after cannabis exposure.

### Evaluation of risk of bias

Risk of bias assessment was conducted independently by two study authors (J.P.& J.H.) using the Risk of Bias in randomized trials (RoB 2) and Risk of Bias in Non-Randomized Studies of Interventions (ROBINS-I) tools [14, 15]. The senior author (AH) settled any discrepancies.

## Results

### Study selection

**Fig. 1:**
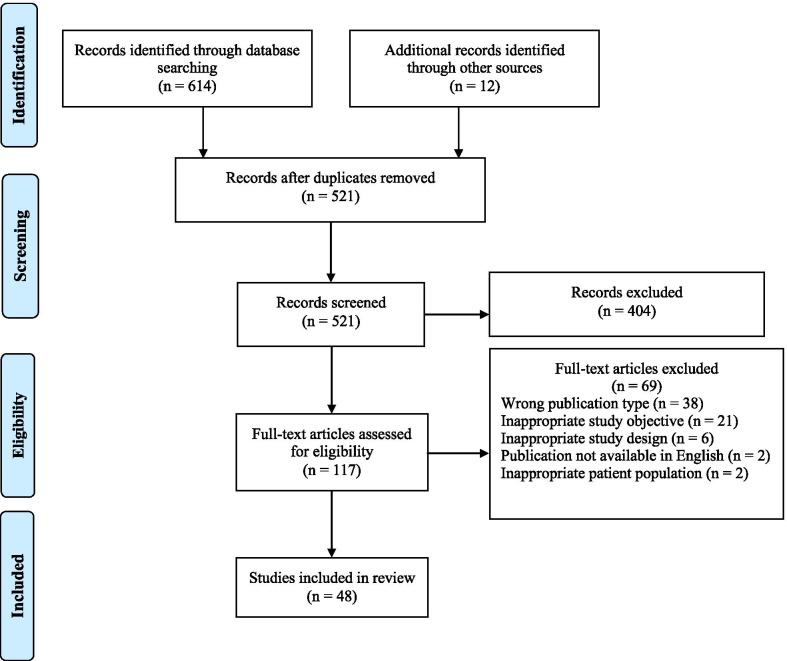
PRISMA flow diagram for review methodology

A study flow diagram is shown in Fig. 1. The comprehensive database search identified 614 articles and 12 additional articles were identified through the hand searching of reference lists. After removal of duplicates, 521 abstracts were screened. Full-text review of 117 articles excluded a further 69 articles. In total, 48 articles met the inclusion criteria and underwent data extraction and analysis.

### Characteristics of included studies

Detailed characteristics of the 48 studies included are described in Table 1. Among the included studies there were: 17 case–control studies, 14 cross-sectional studies, 6 cohort studies, 6 case series, and 5 randomized controlled trials (RCTs). There was significant heterogeneity in study design, objective, and strength of the evidence reported. Quality of the literature ranged from level two to four, as shown in Table 1. The sample size of participants in the included studies ranged from 5 to 29,195. The amount of cannabis consumption was reported by the majority of studies (33). A total of 12 studies expressed cannabis consumption in joint-years, defined as the number of joints smoked per day, multiplied by the duration in years. This ranged from < 1 to 62.1 joint-years. Studies not expressing cannabis consumption in joint-years either failed to report the amount of cannabis use or instead reported frequency or weight of cannabis consumption. A large proportion of participants in the included studies also reported tobacco use in addition to cannabis consumption, ranging from 0 to 89.1%. All included articles reported on at least one otolaryngology related side effect following cannabis exposure.

**Table 1:** Studies reporting risk of otolaryngology-related side effects following cannabis exposure

Study	Study type	Level of evidence	Subspeciality	No. of participants	Sex	Tobacco use	Cannabis use	Main findings
Ahrens and Bressi [16]	Case series	4	Head and neck	5	60% M	80%	100%	5/5 marijuana users developed an erythroplastic lesion, and 2/5 users were determined to have a malignant lesion
Ahrens and Bressi [17]	Case series	4	Head and neck	178	57% M	100%	22%	10/39 tobacco and marijuana users developed oral cancer
Aldington et al. [18]	Case–control	4	Head and neck	394	51% M	53%	14%	The highest tertile of cannabis use (> 8.3 JY) was associated with a nonsignificant increased risk of cancer (RR = 1.6; 95%CI 0.5–52)
Berthiller et al. [19]	Case–control	4	Head and neck	9044	74% M	74%	13%	No association with marijuana use and risk of H&N cancer observed (OR = 0.88; 95%CI 0.67–1.16)
Bhattacharyya et al. [20]	Cross-sectional	4	Head and neck	83	100% M	57%	30%	Overexpression of EGFR onco-proteins is correlated to cannabis smoking (*p* < 0.01)
Bonnet [21]	Cross-sectional	4	Head and neck	39	80% M	N/A	100%	All tested patients were found to have TSH, total T_3_, and free T_4_ levels within the normal range
Brumbach et al. [22]	Cross-sectional	4	Otology	40	48% M	0%	50%	No significant difference was observed in behavioural hearing thresholds between smokers and nonsmokers (p > 0.05)
Carley et al. [23]	RCT	2	Airway	73	71% M	N/A	66%	The proportion of adverse events did not differ between the OSA patients in the placebo and treatment groups (*p* = 0.16)
Cook et al. [24]	Cross-sectional	4	Head and neck	1, 010	100% F	5%	4%	Oral HPV infection were associated with smoking marijuana (*p* = 0.03)
Darling and Arendorf [25]	Cohort	3	Head and neck	579	N/A	71%	46%	Cannabis users did not show greater prevalence of leukoplakia when compared with control groups
Darling et al. [26]	Cohort	3	Head and neck	48	100% M	67%	33%	No significant difference in the effect of cannabis on the epithelial cells of the oral cavity
Darling et al. [27]	Cross-sectional	4	Head and neck	163	N/A	69%	34%	Cannabis smoking significantly increases the prevalence of oral *C. albicans* compared to tobacco only smokers and non-smokers (*p* = 0.022)
Donald [28]	Case series	4	Head and neck	6	100% M	66%	100%	Chronic marijuana use may be a contributor to H&N cancer production in young patients
Feng et al. [29]	Case–control	4	Head and neck	1, 251	69% M	41%	6%	Ever consumption of cannabis was significantly associated with increased nasopharyngeal carcinoma risk (*p* < 0.025)
Gillison et al. [30]	Case–control	4	Head and neck	562	79% M	86%	18%	Marijuana smoking was strongly associated with HPV-16-positive HNSCC in a dose–response relationship (OR = 4.7; 95%CI 1.3–17)
Han et al. [31]	Cross-sectional	4	OtologyRhinologyAirway	29, 195	N/A	19%	39%	Marijuana use increases the occurrence of sinusitis (OR = 1.23; 95%CI 0.99–1.28), but has no effect on sleep apnea (OR = 1.20; 95%CI 0.92–1.56) and tinnitus (OR = 1.14; 95%CI 0.77–1.70)
Hashibe et al. [32]	Case–control	4	Head and neck	2, 252	61% M	65%	54%	No association observed between marijuana use and oral (OR = 1.1; 95%CI 0.8–1.5), pharyngeal (OR = 0.75; 95%CI 0.37–1.5), or laryngeal (OR = 0.93; 95%CI 0.5–1.7) cancer
Henderson et al. [33]	Case series	4	Rhinology	200	100% M	90%	100%	26/200 patients presented with rhinitis and 150/200 patient had symptoms of pharyngitis
Herning et al. [34]	Cross-sectional	4	Head and neck	108	60% M	N/A	69%	Marijuana users that used for > 8 years had lower T_4_ (*p* < 0.01) and higher T_3_ uptake (*p* < 0.05) levels compared to short term marijuana users
Hess et al. [35]	Case–control	4	Head and neck	162	88% M	N/A	43%	HPV-negative patients has higher rates of marijuana use compared to HPV-positive patients (*p* = 0.003)
Kagen et al. [36]	Cross-sectional	4	Head and neck	38	43% M	64%	74%	Marijuana smoking sensitizes patients to Aspergillus
Liang et al. [37]	Case–control	4	Head and neck	981	73% M	73%	27%	Moderate marijuana use (10–20 years) was associated with a reduced risk of HNSCC (OR = 0.52; 95%CI 0.34–0.89)
Liedgren et al. [38]	RCT	2	Otology	30	77% M	N/A	100%	Marijuana has no effect on hearing acuity as assessed by pure tone threshold, speech reception threshold, speech discrimination, and acoustic impedance measurement
Llewellyn et al. [39]	Case–control	4	Head and neck	323	56% M	69%	11%	No significant association between cannabis use and oral cancer (OR = 1.0; 95%CI 0.5–2.2.)
Llewellyn et al. [40]	Case–control	4	Head and neck	144	53% M	69%	13%	No significant association between cannabis use and oral cancer (OR = 0.3; 95%CI 0.1–1.8.)
Malhotra et al. [41]	Cross-sectional	4	Head and neck	5, 280	50%	N/A	55%	Recent marijuana use was not associated with thyroid dysfunction, but was significantly associated with lower levels of TSH (0.344; 95%CI 0.127–0.928)
Marks et al. [42]	Case–control	4	Head and neck	9, 916	71% M	66%	16%	Marijuana use was associated with an elevated risk of oropharyngeal cancer (OR = 1.24; 95%CI 1.06–1.47), and a reduced risk of oral tongue cancer (OR = 0.47; 95%CI 0.29–0.75). Possible association with HPV
Mueller and Wilcox [43]	Cross-sectional	4	Laryngology	35	26% M	29%	40%	Marijuana users did not differ perceptually from nonsmokers and tobacco smokers in vocal pitch, vocal quality, and fundamental frequency; however, users had darker vocal cords on indirect laryngoscopy
Mulheran et al. [44]	RCT	2	Otology	8	100% M	N/A	100%	THC does not appear to have a profound effect on the processing of elementary stimuli by the auditory pathway as assessed by pure tone audiometry
Muller et al. [45]	Case–control	4	Head and neck	289	53% M	70%	48%	Marijuana use was associated with oral HPV detection in HIV-negative patients (OR = 4.0; 95%CI 1.3–12.4), but not HIV-positive patients
Newman et al. [46]	Cross-sectional	4	Head and neck	39	85% M	N/A	51%	Marijuana use was associated with a change of the oral microbiota at the oral pharyngeal site that were more consistent with cancer
Parshad et al. [47]	Case–control	4	Head and neck	56	100% M	0%	100%	Serum T_3_ levels were lower in smokers compared to non-smokers (*p* < 0.05)
Prasad et al. [48]	Case series	4	Airway	17	35% M	N/A	100%	Dronabinol treatment is safe and significantly reduces the apnea–hypopnea index in patients with sleep apnea (*p* = 0.003)
Rosenblatt et al. [49]	Case–control	4	Head and neck	1, 022	71% M	78%	25%	No association observed between marijuana use and OSCC (OR = 0.9; 95%CI 0.6–1.3)
Shah et al. [50]	Case–control	4	Head and neck	1, 000	80% M	27%	1%	Patients with head and neck cancer were more likely to smoke marijuana (OR = 1.6)
Spector [51]	RCT	2	Otology	72	N/A	N/A	100%	No vestibular effect was observed after smoking marijuana
Spector [52]	Cross-sectional	4	Otology	89	56%	N/A	73%	No auditory differences observed between chronic marijuana users and controls, but significant changes in vestibular function seen in chronic marijuana users as assessed by electronystagmography
Stokes et al. [53]	Cohort	3	Rhinology	127	N/A	N/A	N/A	78/128 patients were skin test positive to cannabis, and 22/30 of the patients with positive skin testing has respiratory symptoms consistent with allergic rhinitis
Tashkin et al. [54]	Cohort	3	Airway	299	67% M	69%	93%	Compared to nonsmokers, marijuana smokers did not report a significantly increased percentage of dyspnea
Taylor [55]	Case series	4	Head and neck	10	60% M	60%	70%	7/10 patients with respiratory tract carcinoma were marijuana users
Tennant et al. [56]	Cohort	3	Rhinology	31	100% M	68%	100%	12/31 hashish users presented with symptoms of rhinopharyngitis
Weich et al. [57]	Cross-sectional	4	Otology	17	N/A	N/A	59%	7/17 users reported hearing loss; 6/17 users reported dizziness; 6/17 users reported tinnitus
Winton-Brown et al. [58]	RCT	2	Otology	14	100% M	N/A	100%	THC attenuated activation in the primary and secondary auditory regions of the brain bilaterally relative to placebo (*p* = 0.0006)
Xie et al. [59]	Case–control	4	Head and neck	879	72% M	52%	8%	A higher rate of oropharynx cancer was observed within marijuana users (*p* < 0.0001); marijuana users had a higher rate of HPV-16 positive oropharyngeal cancer (*p* = 0.002)
Zhang et al. [60]	Case–control	4	Head and neck	349	63% M	72%	12%	The risk of HNSCC is increased with marijuana use compared to no use in a dose–response relationship (OR = 2.6; 95%CI 1.1–6.6)
Zhang et al. [61]	Case–control	4	Head and neck	284	64% M	76%	11%	Marijuana use combined with environmental tobacco smoke exposure is a potential risk factor for HNSCC (OR = 7.1; 95%CI 1.5–34.5)
Zhang et al. [62]	Cohort	3	Head and neck	94	81% M	24%	50%	No survival difference between HPV-related OPSCC marijuana users and non-users in 2-year and 5-year survival (*p* = 0.400)
Zuskin et al. [63]	Cross-sectional	4	AirwayRhinology	190	24% M	34%	100%	Male and female hemp works experienced a significantly higher prevalence of sinusitis compared to controls; female works also experienced a higher prevalence of dyspnea

JY, Joint years; EGFR, epidermal growth factor receptor; OSA, obstructive sleep apnea; HPV, human papillomavirus; HNSCC, head and neck squamous cell carcinoma; OSCC, oral squamous cell carcinoma; OPSCC, oropharyngeal squamous cell carcinoma; OR, odds ratio; N/A, not applicable

### Subspecialty synthesis

**Fig. 2:**
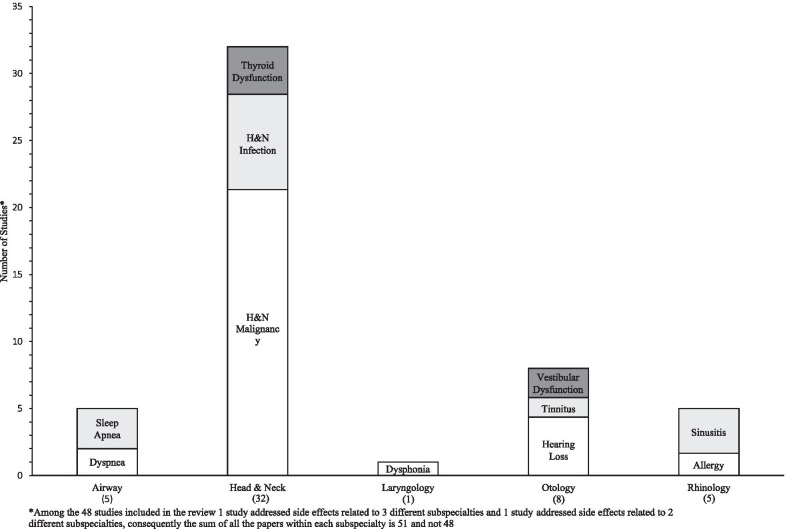
Number of studies reporting the effect of cannabis on the risk of otolaryngology-related side effects

A wide variety of otolaryngology subspecialties were represented (Fig. 2). Below is a synthesis of the results and highlights of themes, trends, and gaps categorized by subspecialty.

#### Head and neck

The most studies were published in head and neck (H&N) (n = 32), with the majority evaluating cannabis’ association with H&N malignancy (n = 24) [16–20, 25, 26, 28–30, 32, 37, 39, 40, 42, 45, 46, 49, 50, 55, 59–62]. Eleven studies reported an increased risk of H&N malignancy following cannabis exposure. Conversely, 12 studies reported no change in risk and two studied reported a decreased risk of H&N cancer following cannabis use. In general, cannabis exposure (via smoking) was associated with increased risk of oropharyngeal carcinoma [30, 42, 59], while oral cavity carcinoma risk was unaffected [32, 39, 40, 42]. This effect appeared to be dose dependant in nature, where low-moderate use had a reduced effect on carcinoma development [37, 42], while higher lifetime use associated a greater risk of malignancy [30, 60].

Eight studies described an association between cannabis and H&N infection, for example, human papilloma virus (HPV) and aspergillus [24, 26, 27, 30, 35, 36, 45, 59]. Three studies reported a positive association between HPV and cannabis exposure [24, 30, 59], while three studied reported no association [26, 35, 45]. Four studies identified the effect of cannabis use on thyroid function [21, 34, 41, 47]. One study reported no association [21] and three study showed a significant association [34, 41, 47].

Overall, forming a firm conclusion on the effects of cannabis in H&N may be challenging, as many studies reported opposing findings. For example, Berthiller et al. described no association between cannabis use and the risk of H&N cancer [19]. However, Zhang et al. provided evidence for a strong dose–response pattern between cannabis use and the risk of H&N malignancy, while also reporting a synergistic effect of cannabis use and cigarette smoking on cancer risk [61]. This area was an unresolved knowledge gap in this scoping review.

Level of evidence: Mostly 4.

#### Otology

The second most studied subspeciality area was otology, where eight studies evaluated cannabis’ association with hearing loss (n = 6), vestibular dysfunction (n = 3) and tinnitus (n = 2) [22, 31, 38, 44, 51, 52, 57, 58]. Please note that one study may have addressed several otologic side effects. Exposure to cannabis was also shown to promote changes in the auditory pathway and alter the function of outer hair cells, while chronic use of cannabis at high doses showed significant changes in vestibular function [22, 52, 57, 58]. There were several excellent basic science otology studies, however, they were excluded because the goal was to keep a clinical focus for this review. Four of the eight otology studies were randomized controlled trials, so the level of evidence was highest for this subspecialty area.

Level of Evidence: 2 and 4.

#### Rhinology

In rhinology, all five studies reported positive association between sinusitis (n = 4) and allergic rhinitis (n = 2) [31, 33, 53, 56, 63]. One rhinology study evaluated both diagnoses [56]. Cannabis smoking was associated with sinusitis in a large retrospective analysis of the US National Survey on Drug Use and Health database (n = 29,195), which was the largest sample size of all the studies in this scoping review [31]. Stokes et al. provided evidence to support cannabis as a clinically relevant aeroallergen on multi-test skin testing [53]. There was the least controversy in this subspecialty area.

Level of Evidence: 3 and 4.

#### Airway

Five studies reported cannabis and its associations with upper airway issues, including sleep apnea (n = 3) and dyspnea (n = 2) [23, 31, 48, 54, 63]. Four studies described no change in risk following cannabis exposure and one study reported an increased risk. Primary evidence supports cannabinoids as a promising and safe treatment option for OSA [48]. One of the randomized controlled trials also concluded that there was no significant difference in adverse events between patients who were treated with cannabis and placebo [23]. A recent position statement by the American Academy of Sleep Medicine, however, recommended against medical cannabis for the treatment of OSA due to unreliable delivery methods and insufficient evidence of effectiveness, tolerability, and safety [64].

Level of Evidence: 2, 3, and mostly 4.

#### Laryngology

Only one study in laryngology explored the effect of cannabis on the voice [43]. This older study published in 1980 reported that marijuana users did not differ perceptually from non-smokers and cigarette smokers, and objective voice analysis was also similar. Darker vocal folds were seen on laryngoscopy. This subspecialty was the weakest with the largest knowledge gap.

Level of evidence: 4.

### Risk of bias assessment

Overall risk of bias for all RCTs was determined to be high, with some concerns in two studies, and low in the remaining three randomized studies (“Appendix B”). Bias in nonrandomized trials was moderate in 21 studies, serious in 12, and critical in 10 studies (“Appendix C”). The major sources of bias among serious and critical studies were due to selection bias, deviations from intended interventions, confounding and inappropriate methods to control for measured confounders. The majority of nonrandomized studies demonstrated appropriate measurement of outcomes and reporting of outcome data.

## Discussion

It is evident from this review that the Otolaryngology cannabis literature is limited but evolving. The included reports demonstrate that unintended side effects in otolaryngology cover a large spectrum of physiology and pathology. These included: tinnitus, vertigo, hearing loss, infection, malignancy, sinusitis, allergic rhinitis, thyroid dysfunction, and dyspnea.

A number of challenges were observed in the included studies. Many studies contained small sample sizes with methodological errors, substantial heterogeneity of study types and varied outcome measures. A lack of standardization in the reporting of quantity, method of consumption, and length of time of cannabis use was common. Similar to cigarette smoking, several studies attempted to use pack-years, but this was inconsistent. Standard medical practice depends on patient self-reporting cannabis use, which leads to recall bias in the literature. Other studies inconsistently used measures to quantify the amount of cannabis in their experimental protocol (e.g. weight, puffs, joints). This did not always control for the type of cannabis, which has the potential to alter expected results substantially. The included studies were published over several decades, however an improvement in the quality of papers were seen over time. Five recent studies were RCTs, suggesting that as cannabis gains widespread acceptance, the medical community recognizes the need for robust evidence. Lastly, challenges arise when studying cannabis consumption with concomitant tobacco and alcohol use. The carcinogenic effects of cannabis, when smoked, is certainly another area of relevance. Although these factors were controlled for by some studies, they will remain confounders until their relationship can be explored entirely.

The medical use of cannabis is legal in 33 US states, and since 2012, 11 states have legalized recreational use of the drug [65]. Conservative estimates project legal cannabis sales to reach $30 billion by 2025, driven mainly by increased legalization of the drug across the country [66]. This swift legalization of cannabis and growth of related industry is likely to lead to the assumption among the general public that cannabis has an adequate safety profile, analogous to the perceived safety, and subsequent increase in consumption of alcohol following prohibition in the 1930’s [67, 68]. Anecdotally, cannabis has been used to treat a large variety of conditions ranging from acute pain and psychiatric diagnoses, to chronic inflammatory conditions [69]. However, only three indications have substantial evidence demonstrating cannabis as an effective treatment alternative. These include chronic pain in adults, oral antiemetic therapy in chemotherapy-induced nausea and vomiting, and an improvement in patient reported spasticity in multiple sclerosis [70]. Outside of these few indications, there is limited and conflicting evidence to support the effectiveness of cannabis as a primary treatment for most medical conditions. This trend is similarly demonstrated in our review, with a disparity in consistency of the reported side effects. As we learn more about cannabis, we will likely understand that there are many more interactions and side effects associated with varying amounts of use. Many of these effects will likely not be observed for years, when evidence on chronic use begin to emerge. Conversely, there is the possibility for positive uses that remain to be realized. Given this, it would be appropriate for all health practitioners to develop and incorporate a better understanding of this drug into their practice as the literature continues to evolve.

There are obvious gaps in the knowledge of how cannabis affects otolaryngology, but this also suggests extensive opportunities for further research. Every subspecialty of Otolaryngology remains a potential area for further study. Presently, the areas of H&N oncology, otology and allergy demonstrate convincing effects of cannabis, making them easy study prospects. Furthermore, the quantification of cannabis use over time and understanding the potential risks may lead to harm reduction campaigns, notably with young and heavy users.

A small number of recent systematic reviews have studied cannabis in subspecialties within Otolaryngology, such as laryngology and H&N oncology. Meehan-Atrash et al. in [4], assessed the association between inhaled cannabis and voice disorders. Similar to our review, the group only identified a single clinical study specific to voice. De Carvalho et al. in [10] performed a meta-analysis to establish the relationship between marijuana and H&N cancer. This group reported no association between lifetime marijuana use and H&N malignancy. Although these researchers were able to reach a conclusion on H&N cancer risk, the findings of our review have dissuaded us from doing the same as we believe that much of the H&N oncology literature is divided and still in its infancy. Furthermore, de Carvalho et al. (2015) limited their meta-analysis to case–control studies. Considering the cannabis literature has evolved significantly since 2015, we believe that higher-quality studies, including longitudinal studies, are needed to reach a clear consensus. A narrative review on the evidence for the use of cannabis in otolaryngology was recently completed by Valentino and Mckinnon. However, they did not adhere to PRISMA guidelines and perform a scoping review of the literature [9]. They did not use the Oxford Centre for Evidence-Based Medicine Levels of Evidence or perform a risk of bias assessment to evaluate the quality of the studies.

The strengths of this scoping review included the rigorous PRISMA methodology, comprehensive peer reviewed search strategy, and the inclusion of a fair number of reports. This was an ideal methodology to study this topic, as scoping reviews are useful for evaluating emerging evidence. Broad areas can be examined to identify knowledge gaps and show how research is currently being conducted in this field, helping to define more precise questions. Recognizing the heterogeneity of the literature, the choice to use a scoping review methodology allowed for data synthesis, hypothesis generation and will serve as a precursor for systematic reviews and future study in this early field. Lastly, this review was the first to group unintended side effects to each subspecialty, and attempt to define a preliminary risk profile of cannabis (i.e. Head and neck malignancy).

There were some limitations. This review was limited to English language studies only. Case studies with less than five patients were excluded to ensure that only higher-quality studies were included. This decision may have excluded very rare side effects. Furthermore, given the multiple methods of cannabis consumption, the severity and degree of side effects may vary. This will likely be overcome as the Cannabis literature becomes more robust. Basic science studies were excluded to maintain a clinical focus in this review. The study was limited to the adult population because most jurisdictions require a legal age limit to consume cannabis.

## Conclusion

This scoping review was undertaken to better understand the safety profile of cannabis and its potential for unintended side effects related to Otolaryngology. Although the quality of evidence in the included reports was lacking, it has provided an overview of the available literature, potential pitfalls or challenges to study design, and was hypothesis-generating. This review indicated that cannabis use is associated with several side effects, including hearing loss, H&N malignancy, and HPV infection. However, much of the H&N literature remains divided on the actual risk of cannabis use. Significant opportunities exist for the otolaryngology community to better investigate the therapeutic effects of cannabis with high-quality studies, particularly on the risk of long-term use and harm reduction. As the rapidly evolving cannabis market continues to grow, clinicians will be expected to guide and counsel patients considering medical or recreational use, and it is essential that they have access to high-quality objective evidence.

## Data Availability

The datasets used and/or analysed during the current study are either included in this published article or are available from the corresponding author on reasonable request.

## Appendix A: Search strategies for MEDLINE, EMBASE, CINAHL, and CENTRAL

### MEDLINE search strategy

1. Otolaryngology/
2. Sinusitis/
3. Rhinitis/
4. Otorhinolaryngologic Diseases/
5. Ear/
6. Nose/
7. Larynx/
8. Palate/
9. Pharynx/
10. Mouth/
11. Tongue/
12. Palatine Tonsil/
13. Thyroid Gland/
14. Neck/
15. Head/
16. Neurotology/
17. Epistaxis/
18. Otitis/
19. Hearing/
20. Tinnitus/
21. Vocal Cords/
22. Vocal Cord Paralysis/
23. "Head and Neck Neoplasms"/
24. otolaryngology.mp. [mp = title, abstract, original title, name of substance word, subject heading word, floating sub-heading word, keyword heading word, organism supplementary concept word, protocol supplementary concept word, rare disease supplementary concept word, unique identifier, synonyms]
25. (head and neck).mp. [mp = title, abstract, original title, name of substance word, subject heading word, floating sub-heading word, keyword heading word, organism supplementary concept word, protocol supplementary concept word, rare disease supplementary concept word, unique identifier, synonyms]
26. 1 or 2 or 3 or 4 or 5 or 6 or 7 or 8 or 9 or 10 or 11 or 12 or 13 or 14 or 15 or 16 or 17 or 18 or 19 or 20 or 21 or 22 or 23 or 24 or 25
27. exp Cannabis/ or exp Cannabinoids/ or exp Marijuana Abuse/ or exp "Marijuana Use"/ or exp Medical Marijuana/ or (((blunt or blunts or pot) adj2 smok*) or bhang or bhangs or cannabi* or cannibinoid* or cesamet or dexanabinol or dronabinol or ganja or ganjas or "hash oil*" or hashish* or hemp or mari?uana* or marinol or nabilone or nabiximol* or sativex or tetrahydrocannabinol* or THC).mp.
28. 26 and 27

### EMBASE search strategy

1. Sinusitis/
2. Otolaryngology/
3. Rhinitis/
4. Otorhinolaryngologic Diseases/
5. Ear/
6. Larynx/
7. Palate/
8. Nose/
9. Pharynx/
10. Mouth/
11. Tongue/
12. Palatine Tonsil/
13. Thyroid Gland/
14. Neck/
15. Head/
16. Neurotology/
17. Epistaxis/
18. Otitis/
19. Hearing/
20. Tinnitus/
21. Vocal Cords/
22. Vocal Cord Paralysis/
23. "Head and Neck Neoplasms"/
24. otolaryngology.mp.
25. (head and neck).mp.
26. 1 or 2 or 3 or 4 or 5 or 6 or 7 or 8 or 9 or 10 or 11 or 12 or 13 or 14 or 15 or 16 or 17 or 18 or 19 or 20 or 21 or 22 or 23 or 24 or 25
27. cannabis smoking/ or medical cannabis/ or cannabis/ or "cannabis use"/ or Cannabis sativa/ or cannabis derivative/
28. cannabis/
29. cannabinoid/
30. cannabinoid/
31. hash.mp.
32. dronabinol/
33. tetrahydrocannabinol/
34. 27 or 28 or 29 or 30 or 31 or 32 or 33
35. 26 and 34

### CINAHL search strategy

1. (MH "Otorhinolaryngologic Diseases + ")
2. (MH "Otorhinolaryngologic Neoplasms + ")
3. (MH "Otorhinolaryngology and Head-Neck Nursing")
4. “otolaryngology”
5. (MH "Sinusitis + ")
6. (MH "Rhinitis + ")
7. (MH "Rhinosinusitis")
8. (MH "Larynx + ")
9. (MH "Head and Neck Neoplasms + ")
10. (MH "Squamous Cell Carcinoma of Head and Neck")
11. (MH "Tinnitus")
12. (MH "Vocal Cord Paralysis + ")
13. (MH "Vocal Cord Dysfunction")
14. (MH "Otitis + ")
15. (MH "Thyroid Diseases + ")
16. (MH "Hearing Disorders + ")
17. 1 or 2 or 3 or 4 or 5 or 6 or 7 or 8 or 9 or 10 or 11 or 12 or 13 or 14 or 15 or 16
18. (MH "Cannabis")
19. (MH "Medical Marijuana")
20. 18 or 19
21. 17 and 20

### CENTRAL search strategy

1. MeSH descriptor: [Otolaryngology] explode all trees
2. MeSH descriptor: [Head and Neck Neoplasms] explode all trees
3. MeSH descriptor: [Otorhinolaryngologic Diseases] explode all trees
4. MeSH descriptor: [Rhinitis] explode all trees
5. MeSH descriptor: [Sinusitis] explode all trees
6. 1 or 2 or 3 or 4 or 5
7. MeSH descriptor: [Cannabis] explode all trees
8. 6 and 7

## Appendix B: Risk of bias assessment (Rob 2): randomized controlled trials

**Table:**

Study	Risk of bias arising from the randomization process	Risk of bias due to deviations from the intended intervention	Risk of bias due to missing outcome data	Risk of bias in measurement of the outcome	Risk of bias in selection of the reported result	Overall risk of bias
Carley et al. [23]	Low	Low	Low	Low	Low	Low
Liedgren et al. [38]	Some concerns	High	Some concerns	High	Low	High
Mulheran et al. [44]	Low	Low	Low	Low	Low	Low
Spector [51]	Some concerns	Some concerns	Low	Low	Low	Some concerns
Winton-Brown et al. [58]	Some concerns	Low	Low	Low	Low	Low

## Appendix C: Risk of bias assessment (ROBINS-I): non-randomized studies

**Table:**

Study	Yr	Bias due to confounding	Bias in selection of participants into the study	Bias in classification of interventions	Bias in deviations from intended Interventions	Bias due to missing data	Bias in measurement of outcomes	Bias in selection of the reported result	Overall bias
Ahrens [16]	2005	Critical	Critical	NI	NI	Low	Serious	Low	Critical
Ahrens [17]	2007	Critical	Serious	Serious	Low	Low	Moderate	Low	Critical
Aldington [18]	2008	Low	Moderate	Low	Low	Low	Moderate	Moderate	Moderate
Berthiller [19]	2009	Low	Moderate	Low	Low	Low	Moderate	Moderate	Moderate
Bhattacharyya [20]	2015	Serious	Moderate	Low	Low	Low	Moderate	Moderate	Serious
Bonnet [21]	2013	Serious	Moderate	Low	NI	Moderate	Moderate	Low	Serious
Brumbach [22]	2019	Low	Moderate	Low	Low	Low	Low	Low	Moderate
Cook [24]	2014	Low	Moderate	Low	Low	Moderate	Moderate	Moderate	Moderate
Darling [25]	1993	Serious	Serious	Low	Low	Moderate	Moderate	Low	Serious
Darling [26]	2002	Serious	Serious	Low	Serious	Low	Moderate	Low	Serious
Darling [43]	1990	Critical	Serious	Serious	NI	Low	Moderate	Low	Critical
Donald [28]	1986	Critical	Serious	NI	NI	Low	NI	NI	Critical
Feng [29]	2019	Low	Moderate	Low	Low	Low	Low	Low	Moderate
Gillison [30]	2008	Low	Moderate	Low	Low	Low	Low	Low	Moderate
Han [31]	2010	Low	Moderate	Low	Low	Low	Moderate	Moderate	Moderate
Hashibe [32]	2006	Low	Low	Low	Low	Low	Low	Low	Moderate
Henderson [33]	1972	Critical	Critical	Serious	NI	NI	Serious	NI	Critical
Herning [34]	2008	Low	Moderate	Low	Low	Low	Low	Low	Moderate
Hess [35]	2014	Low	Moderate	Serious	Low	Low	Low	Low	Serious
Kagen [36]	1983	Critical	Serious	Low	Low	NI	Moderate	Low	Critical
Liang [37]	2009	Low	Moderate	Low	Low	Moderate	Low	Low	Moderate
Llewellyn [39]	2004	Low	Moderate	Low	Low	Low	Low	Low	Moderate
Llewellyn [40]	2004	Low	Moderate	Low	Low	Moderate	Moderate	Low	Moderate
Malhotra [41]	2017	Low	Moderate	Low	Low	Low	Moderate	Low	Moderate
Marks [42]	2013	Low	Moderate	Low	Low	Moderate	Low	Low	Moderate
Mueller [43]	1980	Serious	Serious	Low	Serious	Serious	Low	Low	Serious
Muller [45]	2015	Low	Moderate	Low	Low	Moderate	Low	Low	Moderate
Newman [46]	2019	Critical	Serious	Low	Serious	Low	Moderate	Low	Critical
Parshad [47]	1983	Serious	Serious	Low	NI	Low	Low	Low	Serious
Prasad [48]	2013	Serious	Low	Low	NI	Serious	Low	Low	Serious
Rosenblatt [49]	2004	Low	Moderate	Low	Low	Low	Low	Low	Moderate
Shah [50]	2018	Serious	Serious	Serious	Serious	Low	Low	Low	Serious
Spector [52]	1974	Critical	Serious	Low	Serious	Low	Low	Low	Critical
Stokes [53]	2000	Serious	Serious	Serious	Serious	Low	Serious	Low	Serious
Tashkin [54]	2012	Low	Moderate	Low	Low	Moderate	Moderate	Low	Moderate
Taylor [55]	1988	Critical	Critical	Critical	Serious	Moderate	Serious	Low	Critical
Tennant [56]	1971	Critical	Serious	Critical	Serious	Serious	Serious	Moderate	Critical
Weich [57]	2012	Serious	Serious	Moderate	Serious	Low	Serious	Low	Serious
Xie [59]	2018	Low	Moderate	Low	Low	Low	Low	Low	Moderate
Zhang [60]	1999	Low	Moderate	Low	Low	Low	Low	Low	Moderate
Zhang1 [11]	2000	Low	Moderate	Low	Low	Moderate	Low	Low	Moderate
Zhang [62]	2019	Low	Moderate	Low	Low	Low	Low	Low	Moderate
Zuskin [63]	1990	Serious	Serious	Serious	Serious	Low	Moderate	Low	Serious

NI, no information.

## Abbreviations

HPV: Human papilloma virus
OSA: Obstructive sleep apnea
H&N: Head and neck
RTC: Randomized control trial
